# Preoperative prediction of IDH genotypes and prognosis in adult-type diffuse gliomas: intratumor heterogeneity habitat analysis using dynamic contrast-enhanced MRI and diffusion-weighted imaging

**DOI:** 10.1186/s40644-025-00829-5

**Published:** 2025-02-08

**Authors:** Xingrui Wang, Zhenhui Xie, Xiaoqing Wang, Yang Song, Shiteng Suo, Yan Ren, Wentao Hu, Yi Zhu, Mengqiu Cao, Yan Zhou

**Affiliations:** 1 Department of Radiology, Renji Hospital, Shanghai Jiao Tong University School of Medicine, Shanghai, 200127 China; 2 Department of Radiology, Tongren Hospital, Shanghai Jiao Tong University School of Medicine, Shanghai, 200336 China; 3 MR Research Collaboration Team, Siemens Healthineers Ltd, Shanghai, 200126 China; 4 Department of Radiology, Huashan Hospital, Fudan University, Shanghai, 200040 China; 5 College of Health Science and Technology, Shanghai Jiao Tong University School of Medicine, Shanghai, 200025 China

**Keywords:** Adult-type diffuse glioma, Intratumor heterogeneity, Isocitrate dehydrogenase, Progression-free survival, Dynamic contrast-enhanced perfusion, Diffusion-weighted imaging

## Abstract

**Background:**

Intratumor heterogeneity (ITH) is a key biological characteristic of gliomas. This study aimed to characterize ITH in adult-type diffuse gliomas and assess the feasibility of using habitat imaging based on dynamic contrast-enhanced magnetic resonance imaging (DCE-MRI) and diffusion-weighted imaging (DWI) to preoperatively predict isocitrate dehydrogenase (IDH) genotypes and prognosis.

**Methods:**

Sixty-three adult-type diffuse gliomas with known IDH genotypes were enrolled. Volume transfer constant (K^trans^) and apparent diffusion coefficient (ADC) maps were acquired from DCE-MRI and DWI, respectively. After tumor segmentation, the k-means algorithm clustered K^trans^ and ADC image voxels to generate spatial habitats and extract quantitative image features. Receiver operating characteristic (ROC) curves and area under the curve (AUC) were used to evaluate IDH predictive performance. Multivariable logistic regression models were constructed and validated using leave-one-out cross-validation, and the contrast-enhanced subgroup was analyzed independently. Kaplan-Meier and Cox proportional hazards regression analyses were used to investigate the relationship between tumor habitats and progression-free survival (PFS) in the two IDH groups.

**Results:**

Three habitats were identified: Habitat 1 (hypo-vasopermeability and hyper-cellularity), Habitat 2 (hypo-vasopermeability and hypo-cellularity), and Habitat 3 (hyper-vasopermeability). Compared to the IDH wild-type group, the IDH mutant group exhibited lower mean K^trans^ values in Habitats 1 and 2 (both *P* < 0.001), higher volume (*P* < 0.05) and volume percentage (pVol, *P* < 0.01) of Habitat 2, and lower volume and pVol of Habitat 3 (both *P* < 0.001). The optimal logistic regression model for IDH prediction yielded an AUC of 0.940 (95% confidence interval [CI]: 0.880–1.000), which improved to 0.948 (95% CI: 0.890–1.000) after cross-validation. Habitat 2 contributed the most to the model, consistent with the findings in the contrast-enhanced subgroup. In IDH wild-type group, pVol of Habitat 2 was identified as a significant risk factor for PFS (high- vs. low-pVol subgroup, hazard ratio = 2.204, 95% CI: 1.061–4.580, *P* = 0.034), with a value below 0.26 indicating a 5-month median survival benefit.

**Conclusions:**

Habitat imaging employing DCE-MRI and DWI may facilitate the characterization of ITH in adult-type diffuse gliomas and serve as a valuable adjunct in the preoperative prediction of IDH genotypes and prognosis.

**Clinical trial number:**

Not applicable.

**Supplementary Information:**

The online version contains supplementary material available at 10.1186/s40644-025-00829-5.

## Background

Gliomas are the most prevalent and aggressive primary brain tumors in adults. Current treatment strategies face challenges due to intratumor heterogeneity (ITH), which plays a critical role in malignant progression and resistance to therapy [1, 2]. Gliomas exhibit complex spatial variations in gene expression, histopathology, and microstructure, forming multiple independent habitats influenced by diverse microenvironmental pressures and cellular phenotypes [3, 4]. In adult-type diffuse gliomas, isocitrate dehydrogenase (IDH) gene mutation is associated with lower malignancy and a more favorable prognosis [5]. Preoperative differentiation of IDH mutations is of great significance for prognostic assessment, personalized treatment planning, and clinical decision-making in patients [6]. Genetically, heterogeneity in IDH expression has been found to be correlated with variations in intratumor blood perfusion and vascular permeability [7, 8]. Additionally, glioma malignancy is closely related to tumor cellularity, quantified by cell density and nuclear-to-cytoplasmic ratio, which can also be reflected in the diffusion capacity of water molecules within the tumor tissue [9].

Although histological and molecular analyses are essential for glioma diagnosis, they are often limited by sampling bias and inaccuracies due to the failure to account for tumor spatial heterogeneity [10]. Moreover, repeated and multiple invasive sampling is not advisable. Tumor imaging analysis methods have been widely adopted to assess ITH. However, current mainstream methods, such as histogram analysis or radiomics, predominantly depend on global voxel features within automatically or manually defined tumor volume of interest (VOI), failing to capture subregional characteristics and therefore inadequately characterizing ITH [4, 11–13]. There is a pressing need for non-invasive methods to accurately assess ITH and identify critical subregions within gliomas that could guide biopsy sampling or individualized treatment.

Habitat imaging, an emerging imaging post-processing technique, has shown its potential in exploring tumor ITH in recent years. Previous research has highlighted the value of habitat imaging derived from single-modality or multiparametric magnetic resonance imaging (MRI) in investigating ITH in gliomas. Some studies have identified tumor vascular habitats based solely on dynamic susceptibility contrast perfusion-weighted imaging (DSC-PWI) in gliomas [14, 15]. Park et al. clustered image voxels using k-means from DSC-PWI and apparent diffusion coefficient (ADC) to construct spatial habitats in glioblastoma, overcoming a limitation in the measurement of a single quantitative parameter [12, 16]. This data-driven, unsupervised algorithm does not rely on prior assumptions and remains unaffected by brain tissue normalization, disclosing more comprehensive potential [12, 17, 18].

Diffusion-weighted imaging (DWI) and dynamic contrast-enhanced (DCE)-PWI are useful MRI modalities for assessing glioma malignancy. DWI is more widely practiced in the diagnosis and monitoring of brain tumors. The ADC from DWI is a useful index reflecting the diffusion capacity of water molecules, which is closely correlated with tumor cellularity [9]. Additionally, DCE-MRI has shown its potential in the diagnosis, monitoring, and prognosis of gliomas, primarily due to its ability to characterize glioma microvasculature [19, 20]. Notably, the volume transfer constant (K^trans^) derived from DCE-PWI is strongly associated with the blood-brain barrier disruption in gliomas [20]. In this study, we hypothesized that spatial subregions, identified through a joint analysis of cellularity and vasopermeability landscapes, could characterize ITH in gliomas. Therefore, the aim of this study was to assess the feasibility of using preoperative ITH information, derived from habitat imaging of K^trans^ and ADC parametric maps, to predict IDH mutation status and prognosis in adult-type diffuse gliomas.

## Methods

### Patients

**Fig. 1:**
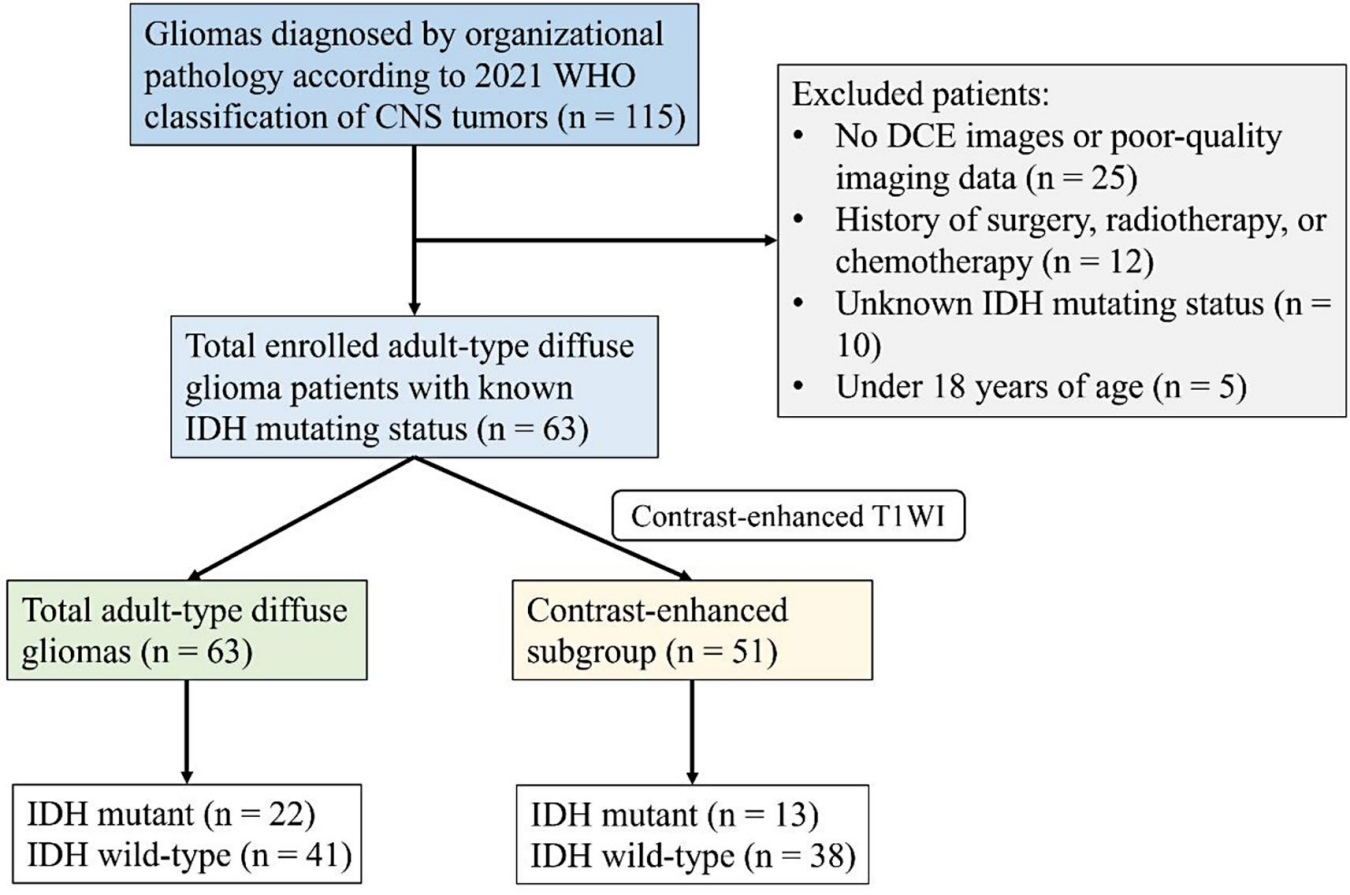
Flow diagram of the patient inclusion process. DCE, dynamic contrast-enhanced; IDH, isocitrate dehydrogenase; T1WI, T1-weighted imaging

This retrospective study was based on a database of 115 consecutive patients with gliomas who were admitted to our hospital between September 2021 and January 2024, with data collected as part of routine clinical care. The study protocol was approved by the local institutional review board and the requirement for informed consent from patients was waived. According to the criteria below, this study enrolled 63 patients with adult-type diffuse gliomas who (1) underwent pre-treatment MR examination including DCE-MRI, with an interval of no more than 10 days; (2) had pathologically confirmed gliomas with results of IDH-1/2 genotype by molecular sequencing; (3) without treatment history before MR scanning, including surgery, radiotherapy, or chemotherapy; (4) with no artifacts of MRI images. Of the all patients, 52 did not meet the inclusion criteria and were excluded: 25 patients had no DCE images or poor-quality imaging data, 12 with a history of treatment, 10 with unknown IDH mutation status because of incomplete molecular or immunohistochemical detection, and 5 were under 18 years of age. Figure 1 shows the patient inclusion process.

### Histopathologic analysis and survival evaluation

According to the 2021 WHO classification of central nervous system (CNS) tumors [1], all IDH mutant diffuse astrocytomas and oligodendrogliomas were considered IDH mutant gliomas, while glioblastomas with wild-type IDH gene were classified as IDH wild-type gliomas. The IDH1 and IDH2 genotypes at the hotspot codons R132 and R172 were determined using the Sanger dideoxy DNA sequencing method. Mutations in either IDH1 or IDH2 were classified as belonging to the IDH mutant group.

We obtained the patients’ progression-free survival (PFS) data from the medical records system, MRI images, and follow-up information. PFS is defined as the time from the patient’s initiation of treatment to tumor progression. Tumor progression on MRI was assessed using the Response Assessment in Neuro-Oncology (RANO) 2.0 criteria [21]. In the newly diagnosed setting, the post-radiotherapy MRI was used as the baseline for comparison with subsequent follow-up scans. The primary measurement was the maximum cross-sectional area of the tumor under stable or increasing doses of corticosteroids, with progression defined as either a 25% increase in the size of the lesions or the appearance of a new measurable lesion; definite clinical deterioration not attributable to causes other than the tumor, or failure to return for evaluation due to deteriorating condition was also considered as progression [22]. All patients were followed up until progression or the study cutoff (January 2025).

### MR image acquisition

MRI acquisition protocol was performed on a 3-T scanner (MAGNETOM Prisma, Siemens Healthcare) with a 20-channel-array head coil. All MRI examinations included unenhanced and gadolinium-based contrast-enhanced (CE) T1-weighted imaging (T1WI), fast spin-echo T2-weighted imaging (T2WI), T2-weighted fluid-attenuated inversion recovery (FLAIR), DWI, and DCE-PWI. For DWI, we performed a single shot echo-planar sequence in the axial plane with diffusion gradient encoding of b = 0 and 1000 s/mm^2^. DCE-MRI images were obtained with a three-dimensional gradient-echo T1-weighted sequence. A bolus injection of 0.1 mmol/kg gadobutrol (Gadovist, 15 ml: 9.0708 g, Bayer) was administered at 2 ml/second by an MRI-compatible power injector (no pre-bolus administration) during the DCE scanning. The injection was initiated at the third phase of the scanning sequence and followed by injecting 15 ml saline at the same injection rate. A total of 30 phases and 840 DCE images were captured with a scanning time of 4 min 42 s. All images were captured with complete tumor volume coverage and the same slice orientation. The parameters for all scanning sequences are provided in Supplementary Table 1.

### Parametric map reconstruction of K^trans^ and ADC

A pharmacokinetic analysis post-processing platform with dedicated software (Tissue-4D, Siemens Healthineers) was used to generate DCE parametric maps. The K^trans^ maps of each patient were calculated by means of the standard Tofts pharmacokinetic model [23].

ADC images were computed from diffusion gradient encoding of b values of 0 and 1000 s/mm^2^ on DWI.

### VOI delineation

All VOIs were manually delineated using 3D Slicer software (version 5.4.0, https://www.slicer.org/; National Institutes of Health, USA) [24]. Before delineation, all image sequences underwent registration and resample referring to T2-FLAIR for normalization to ensure consistent resolution, spacing, and alignment. Tumor segmentation was performed on T2-FLAIR images, and T1WI, T2WI, and CE-T1WI images were consulted to guide VOI delineation. All VOIs covered the tumor solid tissue, excluding peritumoral edema, necrosis, cysts, and obvious non-tumor macro-vessels. The solid tumor area was defined by the T2-FLAIR high-signal area, and the non-enhanced or mildly enhanced tumor area was determined by the high signal boundary on T2WI [25]; any areas that were ambiguously localized or difficult to determine were excluded. Two radiologists (ZHX and XQW, each with 8 years of experience in neuroimaging) performed the VOI delineation by consensus under the supervision of a senior radiologist (MQC, with 15 years of neuroimaging experience in CNS tumors). None of the radiologists were aware of the histopathologic results.

### Multiparametric spatial habitats and quantitative image features

We applied a cohort-based k-means algorithm to cluster image voxels within each tumor VOI into three subregions and extracted quantitative image features from K^trans^ and ADC maps using FeAture Explorer software (FAE, version 0.5.12, Shanghai Key Laboratory of Magnetic Resonance; https://github.com/salan668/FAE.git) [26]. A cluster number of 3 was chosen, as it was the lowest number of clusters to show differences among the imaging parameters, and the lowest number was preferred to avoid over-parameterization of models [16]. Hence, three clusters representing respective habitats in each tumor were finalized (Supplementary Fig. 1): cluster 1 represented “hypo-vasopermeability and hyper-cellularity habitat” (Habitat 1) with low K^trans^ and ADC value; cluster 2 represented “hypo-vasopermeability and hypo-cellularity habitat” (Habitat 2) with low K^trans^ and high ADC value; cluster 3 represented “hyper-vasopermeability habitat” (Habitat 3) with high K^trans^ value. The quantitative image features included the mean K^trans^ (K^trans^_Mean) and mean ADC (ADC_Mean) values in each habitat, as well as the volume and volume percentage (pVol) of each habitat within tumor VOIs. For comparison, the mean K^tran^ and ADC values within the tumor VOIs were extracted independently.

### Morphological assessment

Morphological assessment of image features was independently performed by two radiologists (ZHX and XQW, each with 8 years of experience in neuroimaging), who were both blinded to the diagnosis and the patients’ clinical information. Morphological readings were completed at a separate time (exceeding 4 weeks later than VOI delineation). We selected morphological features that were frequently-used in clinical tumor evaluation, including tumor location, hemorrhage, necrosis, cyst or cysts, edema, and enhancement category [27]. Tumor location was specified by the geographic epicenter of the lesion. Hemorrhage was defined as any intrinsic focus of high signal on T1WI or low signal on T2WI. Unenhanced areas within the tumor body that were patchy or irregular in shape with T2WI hyperintensity and T1WI hypointensity were regarded as necrosis. Cystic regions where the signal was equivalent to that of cerebrospinal fluid, with marginal enhancement not significant or absent, were considered the presence of cyst or cysts. Edema was defined as the area of T2 hyperintensity around the tumor when the solid boundary of the tumor was clear. If the solid tumor and edema could not be distinguished, the T2 hyperintense areas closer to the adjacent brain tissue and showing hyperintensity on the ADC map were considered edema. The minimum distance from the solid tumor to the adjacent white matter was evaluated in the peritumoral edema region, with a threshold of 1 cm selected referring to previous studies [28]. Contrast agent uptake was categorized into patchy or ringlike, or non/mildly enhanced [27].

To validate the performance of subsequent habitat predictive models in differentiating IDH mutation status in obviously enhanced adult-type diffuse gliomas on CE-T1WI, we independently investigated the enhanced subgroup (Fig. 1), excluding all non/mildly enhanced tumors, using the same methodology for supplementary results.

### Statistical analysis

Statistical analyses were performed using SPSS (version 26, IBM), MedCalc (version 22.001, MedCalc Software Ltd.), and R (version 4.3.3, R Foundation). Quantitative variables were reported as mean ± standard deviation (SD), and categorical variables as percentages. All tests were two-tailed with a default alpha level of 0.05.

The interobserver agreement for morphological assessment of image features between the two radiologists was evaluated using Cohen’s kappa test. The Student’s *t*-test or Mann-Whitney *U*-test compared habitat feature differences between IDH mutant and wild-type groups. IDH predictive performance was evaluated using receiver operating characteristic (ROC) curves and area under the curve (AUC). Logistic regression models, incorporating preoperative clinical and imaging features alongside habitat or tumor VOI features, were developed to predict IDH genotypes. After checking multicollinearity, variables with a *P* value < 0.05 in univariable logistic regression and a variance inflation factor (VIF) < 5 were included in multivariable logistic regression models using stepwise method. Model comparisons were performed using the DeLong test, and model fit was assessed by the Hosmer-Lemeshow test. The leave-one-out cross-validation was used to assess the diagnostic efficacy and stability of models.

To further investigate the prognostic value of tumor habitats, we separately divided IDH mutant and wild-type gliomas into two subgroups using the median of each habitat feature. Kaplan-Meier analysis was performed to identify habitat features associated with PFS, and log-rank tests were used to assess the significance. Cox proportional hazards regression was conducted to calculate the hazard ratio (HR) and its 95% confidence interval (CI) for paired subgroups.

## Results

### Patient characteristics

The demographic, clinical, and pathological characteristics of the patients enrolled in this study are summarized in Table 1. The age in the IDH mutant group was significantly lower than that of the wild-type group (*P* < 0.001). In our dataset, 7 cases were WHO grade II gliomas, while the remaining cases were high-grade gliomas (WHO grade III-IV, accounting for 88.89%). There was no statistically significant difference in gender and tumor size between the two IDH groups (*P* = 0.562 and 0.460, respectively). The median follow-up time was 8.0 months (interquartile range: 3.0–13.0 months). During the follow-up period, 39 patients experienced PFS events, including 6 patients in the IDH mutant group and 33 patients in the IDH wild-type group.

**Table 1:** Demographic, clinical, and morphological characteristics of patients

Characteristics	IDH mutant (*n* = 22)	IDH wild-type (*n* = 41)	*P* value
Sex, n			
Male/female	14/8	23/18	0.562
Age, years			
Mean ± SD (range)	44.9 ± 14.0 (24–72)	61.2 ± 13.5 (28–85)	0.000^*^
WHO Grade, n (%)			0.000^*^
II	7 (31.82%)	0 (0%)	
III	7 (31.82%)	0 (0%)	
IV	8 (36.36%)	41 (100%)	
Tumor size, cm^3^			
Mean ± SD	48.44 ± 37.64	42.72 ± 23.54	0.460
Tumor location, n (%)			
Frontal	15 (68.18%)	12 (29.27%)	0.003^*^
Parietal or occipital	1 (4.54%)	9 (21.95%)	0.145
Temporal or insular	5 (22.73%)	12 (29.27%)	0.767
Other	1 (4.54%)	8 (19.51%)	0.144
Hemorrhage, n (%)	7 (31.82%)	16 (39.02%)	0.571
Necrosis, n (%)	9 (40.91%)	30 (73.17%)	0.012^*^
Cyst or cysts, n (%)	14 (63.64%)	22 (53.66%)	0.446
Edema (> 1 cm), n (%)	9 (40.91%)	25 (60.98%)	0.128
Enhancement category, n (%)			
Patchy enhancement	6 (27.27%)	9 (21.95%)	0.636
Ringlike enhancement	7 (31.82%)	29 (70.73%)	0.003^*^
No/mild enhancement	9 (40.91%)	3 (7.32%)	0.002^*^
Follow-up time, months			
Median (IQR)	12.0 (7.8–15.0)	6.0 (2.0–10.0)	0.013^*^

Values are presented as number (%), mean ± SD, or median (IQR). IDH, isocitrate dehydrogenase; SD, standard deviation; IQR, interquartile range. ^*^Represented a statistical difference (*P* < 0.05)

### Morphological features

For tumor location, the agreement between the two observers was good (κ = 0.83, *P* < 0.01). The IDH mutant gliomas were more likely to occur in the frontal lobe than the wild-type (*P* = 0.003), and the latter occurred relatively randomly in different brain regions. There was significant statistical difference in the incidence of necrosis between the two IDH genotypes (*P* = 0.012). In the IDH mutant group, 13 cases (59.1%) exhibited obvious enhancement on CE-T1WI, as did 38 cases (92.7%) in the IDH wild-type group. A ringlike enhancement mode was more common in the wild-type group (*P* = 0.003). The kappa value for the agreement of necrosis and enhancement pattern judgment between the two evaluators was 0.84 (*P* < 0.01) and 0.91 (*P* < 0.001). The agreement between the two evaluators regarding the assessment of hemorrhage, cyst(s), and edema was good (κ = 0.93, 0.89, and 0.88, respectively, all *P* < 0.001), and no significant differences in these features were found between the two IDH genotypes (all *P* > 0.05).

### Habitat features according to IDH mutation status

**Fig. 2:**
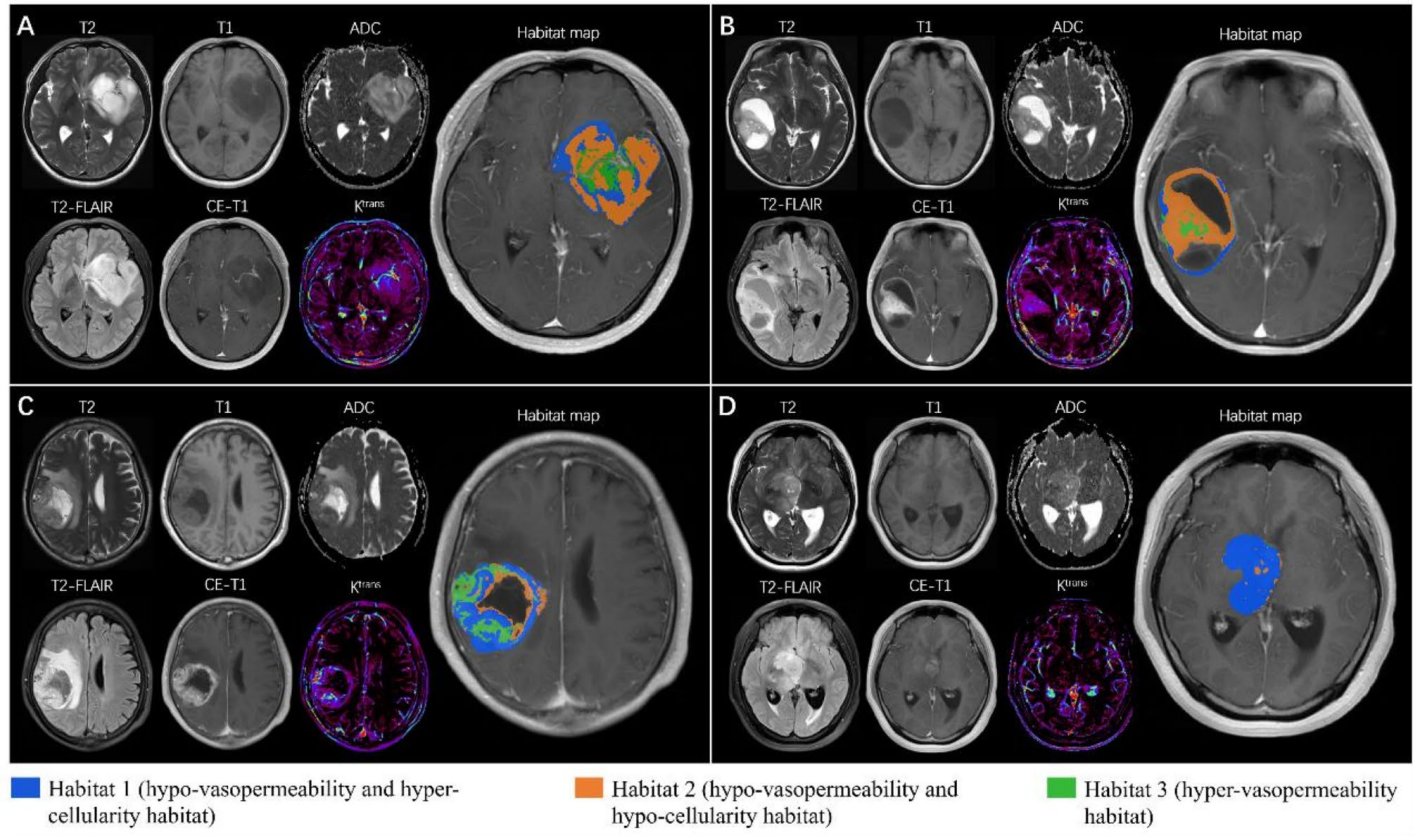
Demonstration of habitat segmentation slices in two patients with IDH mutant and wild-type gliomas. Spatial habitats are labeled with different colors. Contrast-enhanced T1-weighted imaging shows (**A**) a non-enhanced IDH mutant glioma, (**B**) an obviously enhanced IDH mutant glioma, (**C**) an obviously enhanced IDH wild-type glioma, and (**D**) a mildly enhanced IDH wild-type glioma

After clustering ADC and K^trans^ image voxels within the tumor VOI, the three spatial habitats in all gliomas were segmented and labeled with different colors. Figure 2 illustrates the segmented slices in two patients with IDH mutant and wild-type gliomas, respectively.

The statistical results of the quantitative image features for the two IDH genotypes are presented in Table 2. There were significant differences (all *P* < 0.001) in the mean K^trans^ values between IDH mutant and wild-type patients in Habitats 1, 2, and the tumor VOI, with lower values observed in the IDH mutant group. No differences were found in the mean ADC values of the three habitats or tumor VOI between the two IDH genotypes (all *P* > 0.05). Compared with the IDH wild-type, the volume and the pVol of Habitat 2 were higher (*P* < 0.05 and *P* < 0.01, respectively), as those of Habitat 3 were lower (both *P* < 0.001) in IDH mutant gliomas. There was no significant difference in the volume and pVol of Habitat 1 between the two IDH genotype groups (*P* > 0.05).

**Table 2:** Habitat features and tumor VOI-based features in two IDH genotype groups

Features	IDH mutant (*n* = 22)	IDH wild-type (*n* = 41)	*P* value
**Habitat 1**			
ADC_Mean, ×10^− 6^ mm^2^/s	1022.28 ± 91.22	995.98 ± 98.94	0.306
K^trans^_Mean, ×10^− 3^ min^− 1^	34.27 ± 18.24	57.94 ± 18.08	**0.000** ^*^
Volume, mm^3^	26.00 ± 23.21	25.04 ± 18.26	0.858
pVol (%)	52.54 ± 21.44	57.83 ± 22.05	0.363
**Habitat 2**			
ADC_Mean, ×10^− 6^ mm^2^/s	1548.02 ± 79.80	1584.16 ± 116.41	0.199
K^trans^_Mean, ×10^− 3^ min^− 1^	33.03 ± 20.48	57.01 ± 17.10	**0.000** ^*^
Volume, mm^3^	20.77 ± 18.80	11.66 ± 10.54	**0.045** ^*^
pVol (%)	43.57 ± 19.86	28.11 ± 18.51	**0.003** ^*^
**Habitat 3**			
ADC_Mean, ×10^− 6^ mm^2^/s	1194.42 ± 171.81	1163.91 ± 145.55	0.459
K^trans^_Mean, ×10^− 3^ min^− 1^	195.01 ± 27.56	199.59 ± 33.05	0.581
Volume, mm^3^	1.68 ± 2.80	6.01 ± 5.48	**0.000** ^*^
pVol (%)	3.89 ± 4.77	14.06 ± 11.72	**0.000** ^*^
**Tumor VOI**			
ADC_Mean, ×10^− 6^ mm^2^/s	1256.31 ± 168.44	1175.42 ± 182.62	0.090
K^trans^_Mean, ×10^− 3^ min^− 1^	39.94 ± 24.25	77.59 ± 32.89	**0.000** ^*^
Volume, mm^3^	48.44 ± 37.64	42.72 ± 23.54	0.460

Values are presented as mean ± SD. VOI, volume of interest; IDH, isocitrate dehydrogenase; ADC, apparent diffusion coefficient; K^trans^, volume transfer constant; pVol, volume percentage. ^*^Represented a statistical difference (*P* < 0.05)

### Diagnostic performance of habitat features

**Fig. 3:**
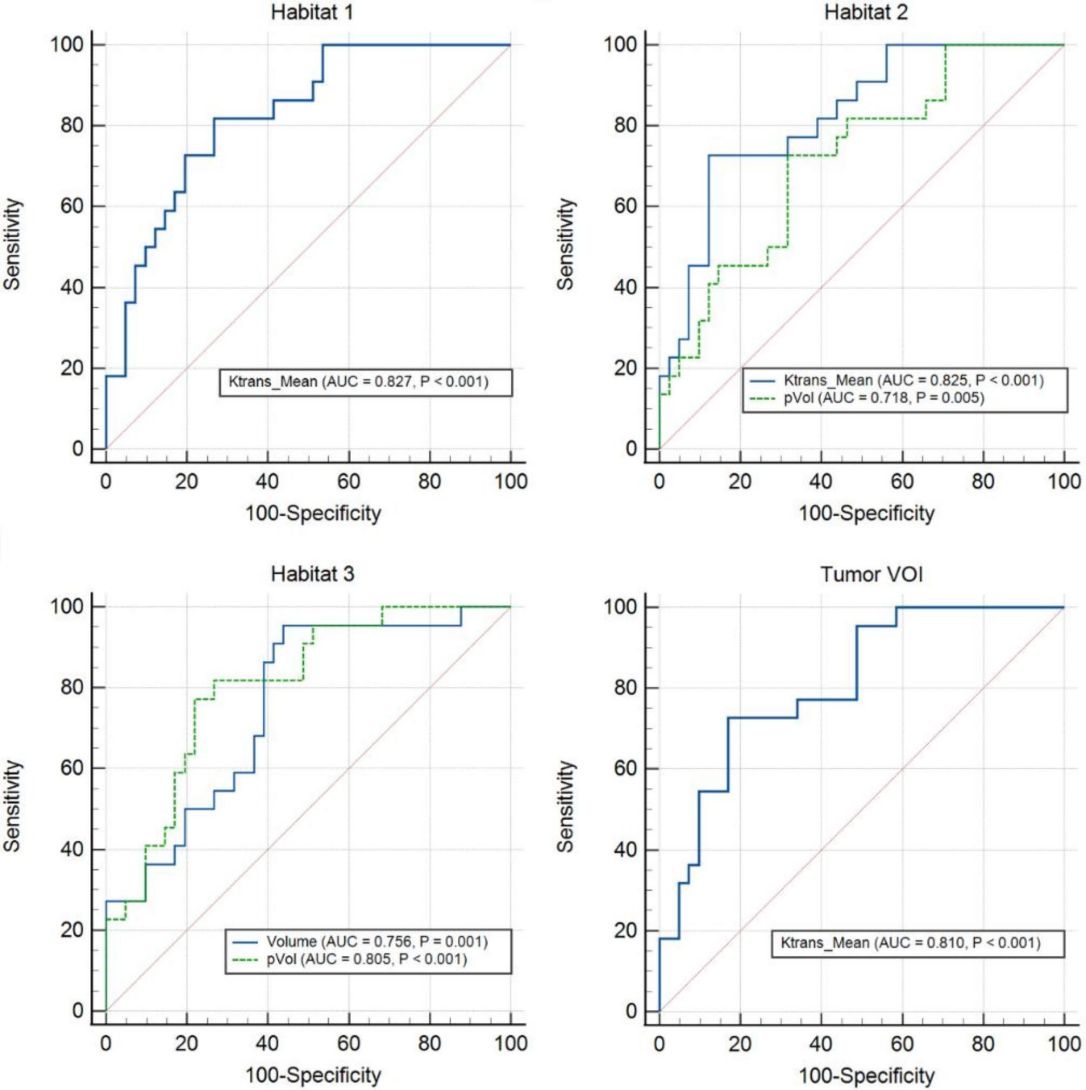
Receiver operating characteristic (ROC) curves of habitat features and tumor volume of interest (VOI)-based features with an area under the ROC curve (AUC) value exceeding 0.7 in discriminating IDH genotypes. Features include K^trans^_Mean (AUC: 0.827, 95% CI: 0.726–0.929, *P* < 0.001) in Habitat 1, K^trans^_Mean (AUC:0.825, 95% CI:0.721–0.929, *P* < 0.001) and pVol (AUC: 0.718, 95% CI: 0.589–0.848, *P* = 0.005) of Habitat 2, Volume (AUC: 0.756, 95% CI: 0.634–0.878, *P* = 0.001) and pVol (AUC: 0.805, 95% CI: 0.696–0.914, *P* < 0.001) of Habitat 3, and K^trans^_Mean (AUC: 0.810, 95% CI: 0.703–0.918, *P* < 0.001) in tumor VOI. Ktrans = K^trans^

ROC curves of all image features with an AUC value exceeding 0.7 are shown in Fig. 3. The mean K^trans^ value in Habitats 1, 2, and the tumor VOI had a good ability to discriminate IDH mutant glioma from its wild-type counterpart (AUC = 0.827, 0.825, and 0.810, all *P* < 0.001). Additionally, the pVol of Habitats 2 and 3, as well as the volume of Habitat 3, could also distinguish IDH mutation from the wild type (AUC = 0.718, 0.805, 0.756, respectively; all *P* < 0.01).

### Multivariable logistic regression analysis in entire cohort and contrast-enhanced subgroup

**Fig. 4:**
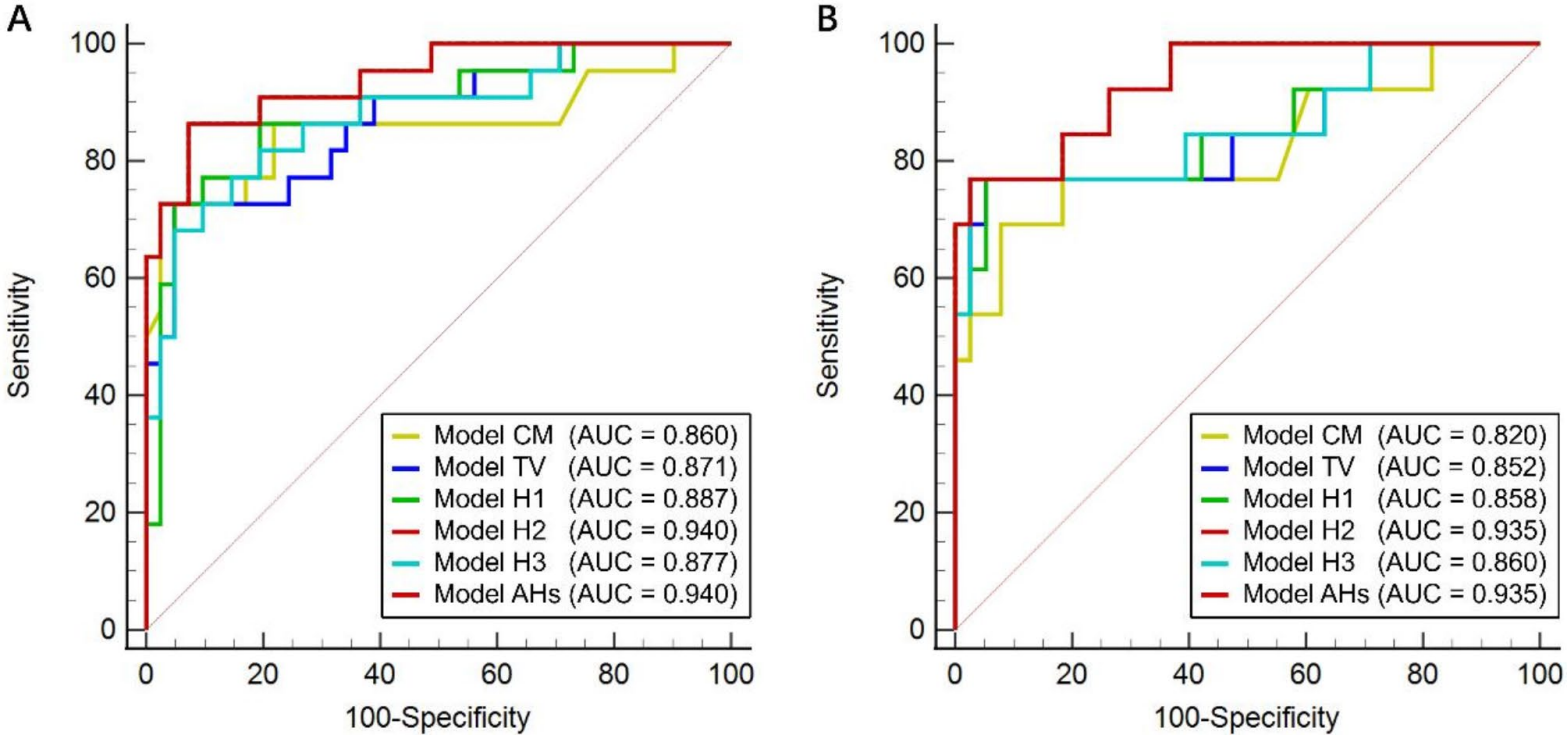
Receiver operating characteristic (ROC) curves of the multivariable logistic regression models for IDH prediction in entire cohort (**A**) and contrast-enhanced subgroup (**B**). Model CM represents the multivariable logistic regression model based solely on clinical and morphological features. Models TV, H1, H2, H3, and AHs correspond to multivariable logistic regression models based on quantitative features from the tumor volume of interest (VOI), Habitats 1, 2, and 3, and all habitats, each incorporating clinical and morphological features

We first established a multivariable logistic regression model (model CM) based on clinical data and morphological features. The final significant variables included age, frontal location, and ringlike enhancement. Model CM attained an AUC value of 0.860 for IDH prediction. Subsequently, multivariable logistic regression analyses incorporating quantitative image features, clinical data, and morphological features were performed. Five comprehensive IDH predictive models (models TV, H1, H2, H3, and AHs based on tumor VOI, Habitats 1, 2, and 3, and all habitats, respectively) were constructed (Table 3). The Hosmer-Lemeshow test confirmed good fit for each model (all *P* > 0.05). The ROC curves of the five models predicting IDH genotypes are presented in Fig. 4A. Among these, model AHs, as well as model H2, demonstrated the best performance, achieving an AUC of 0.940 (95% CI: 0.880-1.000, *P* < 0.001; sensitivity: 86.4%; specificity: 92.7%; see Table 4 for details).

**Table 3:** Univariable and multivariable logistic regression analysis for IDH genotype prediction

Variable	Univariable			Multivariable	
	OR (95% CI)	*P* value	VIF^*^	OR (95% CI)	*P* value
**Model CM**					
Age	0.924 (0.885, 0.965)	0.000	1.228	0.934 (0.891, 0.980)	0.005
Frontal location	5.179 (1.687, 15.893)	0.004	1.104	5.441 (1.313, 22.548)	0.020
Necrosis	0.254 (0.085, 0.759)	0.014	1.284		
Ringlike enhancement	0.193 (0.063, 0.593)	0.004	1.619	0.172 (0.041, 0.717)	0.016
No/mild enhancement	8.769 (2.056, 37.402)	0.003	1.633		
**Model TV**					
Age	0.924 (0.885, 0.965)	0.000	1.235	0.929 (0.884, 0.975)	0.003
Frontal location	5.179 (1.687, 15.893)	0.004	1.477		
Necrosis	0.254 (0.085, 0.759)	0.014	1.287		
Ringlike enhancement	0.193 (0.063, 0.593)	0.004	1.930		
No/mild enhancement	8.769 (2.056, 37.402)	0.003	1.638		
ADC_Mean	1.003 (1.000, 1.006)	0.094			
K^trans^_Mean	0.956 (0.933. 0.980)	0.000	1.809	0.956 (0.930, 0.983)	0.002
Volume	1.007 (0.989, 1.025)	0.455			
**Model H1**					
Age	0.924 (0.885, 0.965)	0.000	1.233	0.931 (0.887, 0.978)	0.004
Frontal location	5.179 (1.687, 15.893)	0.004	1.345		
Necrosis	0.254 (0.085, 0.759)	0.014	1.285	0.259 (0.059, 1.139)	0.074
Ringlike enhancement	0.193 (0.063, 0.593)	0.004	1.807		
No/mild enhancement	8.769 (2.056, 37.402)	0.003	1.665		
ADC_Mean	1.003 (0.997, 1.009)	0.302			
K^trans^_Mean	0.936 (0.903, 0.969)	0.000	1.627	0.938 (0.900, 0.979)	0.003
Volume	1.002 (0.977, 1.029)	0.855			
pVol	0.989 (0.965, 1.013)	0.357			
**Model H2**					
Age	0.924 (0.885, 0.965)	0.000	1.563	0.918 (0.867, 0.972)	0.004
Frontal location	5.179 (1.687, 15.893)	0.004	1.494		
Necrosis	0.254 (0.085, 0.759)	0.014	1.299	0.216 (0.038, 1.217)	0.082
Ringlike enhancement	0.193 (0.063, 0.593)	0.004	1.932		
No/mild enhancement	8.769 (2.056, 37.402)	0.003	1.873		
ADC_Mean	0.997 (0.991, 1.002)	0.198			
K^trans^_Mean	0.940 (0.910, 0.971)	0.000	1.637	0.945 (0.907, 0.984)	0.006
Volume	1.046 (1.005, 1.089)	0.028	2.033		
pVol	1.043 (1.012, 1.074)	0.006	1.908	1.074 (1.020, 1.131)	0.007
**Model H3**					
Age	0.924 (0.885, 0.965)	0.000	1.329	0.924 (0.879, 0.971)	0.002
Frontal location	5.179 (1.687, 15.893)	0.004	1.402		
Necrosis	0.254 (0.085, 0.759)	0.014	1.305		
Ringlike enhancement	0.193 (0.063, 0.593)	0.004	1.921		
No/mild enhancement	8.769 (2.056, 37.402)	0.003	1.638		
ADC_Mean	1.001 (0.998, 1.005)	0.453			
K^trans^_Mean	0.995 (0.978, 1.013)	0.576			
Volume	0.759 (0.621, 0.926)	0.007	3.378		
pVol	0.853 (0.767, 0.948)	0.003	3.695	0.840 (0.742, 0.951)	0.006
**Model AHs**					
Age	0.924 (0.885, 0.965)	0.000	1.563	**0.918 (0.867**,** 0.972)**	**0.004**
Frontal location	5.179 (1.687, 15.893)	0.004	1.553		
Necrosis	0.254 (0.085, 0.759)	0.014	1.299	0.216 (0.038, 1.217)	0.082
Ringlike enhancement	0.193 (0.063, 0.593)	0.004	2.073		
No/mild enhancement	8.769 (2.056, 37.402)	0.003	1.873		
K^trans^_Mean_Habitat 1	0.936 (0.903, 0.969)	0.000			
K^trans^_Mean_Habitat 2	0.940 (0.910, 0.971)	0.000	2.317	**0.945 (0.907**,** 0.984)**	**0.006**
Volume_Habitat 2	1.046 (1.005, 1.089)	0.028	2.033		
pVol_Habitat 2	1.043 (1.012, 1.074)	0.006	1.912	**1.074 (1.020**,** 1.131)**	**0.007**
Volume_Habitat 3	0.759 (0.621, 0.926)	0.007			
pVol_Habitat 3	0.853 (0.767, 0.948)	0.003	2.298		

Model CM represents the multivariable logistic regression model based on clinical and morphological data. Models TV, H1, H2, and H3 are extended models based on the tumor volume of interest (VOI) and Habitats 1, 2, and 3, respectively. Model AHs is an extended model based on all habitats. IDH, isocitrate dehydrogenase; OR, odd ratio; CI, confidence interval; VIF, variance inflation factor; ADC, apparent diffusion coefficient; K^trans^, volume transfer constant; pVol, volume percentage. ^*^Variables with a value of less than 5 for this metric were finally retained before being included in the multivariable regression model

**Table 4:** Comparison of diagnostic performance of IDH predictive models in entire cohort and contrast-enhanced subgroup

Models	AUC (95% CI)	Sensitivity (%)	Specificity (%)	Accuracy (%)	*P* value	*P* value of Delong test^*^	Goodness of fit test^#^	
							χ^2^	*P* value
**Entire cohort**								
Model CM	0.860 (0.741, 0.978)	72.7	97.6	88.9	0.000	0.044	12.947	0.114
Model TV	0.871 (0.775, 0.967)	72.7	95.1	85.7	0.000	0.019	3.124	0.926
Model H1	0.887 (0.795, 0.979)	72.7	95.1	85.7	0.000	0.029	5.932	0.655
Model H2	0.940 (0.880, 1.000)	86.4	92.7	90.5	0.000	1.000	5.173	0.739
Model H3	0.877 (0.781, 0.973)	68.2	95.1	84.1	0.000	0.026	7.327	0.502
**Model AHs**	**0.940 (0.880**,** 1.000)**	**86.4**	**92.7**	**90.5**	**0.000**	**-**	**5.173**	**0.739**
**Contrast-enhanced subgroup**								
Model CM	0.820 (0.664, 0.975)	69.2	92.1	84.3	0.001	0.043	11.251	0.188
Model TV	0.852 (0.707, 0.997)	76.9	94.7	90.2	0.000	0.057	8.181	0.416
Model H1	0.858 (0.720, 0.996)	76.9	94.7	86.3	0.000	0.051	7.450	0.489
Model H2	0.935 (0.862, 1.000)	76.9	97.4	90.2	0.000	1.000	8.411	0.394
Model H3	0.860 (0.719, 1.000)	76.9	97.4	90.2	0.000	0.076	12.472	0.131
**Model AHs**	**0.935 (0.862**,** 1.000)**	**76.9**	**97.4**	**90.2**	**0.000**	**-**	**8.411**	**0.394**

Model CM represents the multivariable logistic regression model based solely on clinical and morphological features. Models TV, H1, H2, H3, and AHs correspond to multivariable logistic regression models based on quantitative features from the tumor volume of interest (VOI), Habitats 1, 2, and 3, and all habitats, each incorporating clinical and morphological features. IDH, isocitrate dehydrogenase; AUC, area under curves; CI, confidence interval; VOI, volume of interest. ^*^Model comparison with model AHs. ^**#**^Hosmer-Lemeshow test

In the multivariable logistic regression analysis of model AHs, age (odds ratio [OR]: 0.918, 95% CI: 0.867–0.972, *P* = 0.004), K^trans^_Mean in Habitat 2 (OR: 0.945, 95% CI: 0.907–0.984, *P* = 0.006), and pVol of Habitat 2 (OR:1.074, 95% CI: 1.020–1.131, *P* = 0.007) were identified as significant predictors of IDH mutation status. The Delong test indicated that the AUC of model AHs was significantly higher than those of the other models (all *P* < 0.05), except for model H2. Leave-one-out cross-validation of both model AHs (cvAHs) and model H2 (cvH2) showed superior performance (AUC for model cvAHs: 0.948, 95% CI: 0.890-1.000, *P* < 0.001; AUC for model cvH2: 0.935, 95% CI: 0.864-1.000, *P* < 0.001; shown in Supplementary Fig. 2).

The contrast-enhanced subgroup, as shown in Fig. 1, included 51 patients (13 IDH mutant gliomas and 38 IDH wild-type gliomas) with obviously enhanced tumors on CE-T1WI. Statistical results of quantitative image features for this subgroup are in Supplementary Table 2, while the univariable and multivariable logistic regression analyses are summarized in Supplementary Table 3. Among the multivariable logistic regression models, both models AHs and H2 demonstrated better performance in IDH genotype prediction, with a consistent AUC of 0.935 for both (95% CI: 0.862-1.000, *P* < 0.001; sensitivity: 76.9%; specificity: 97.4%; as shown in Table 4; Fig. 4B). Significant variables in these two models for IDH prediction remained age (OR: 0.924, 95% CI: 0.866–0.985, *P* = 0.015), K^trans^_Mean in Habitat 2 (OR: 0.953, 95% CI: 0.911–0.998, *P* = 0.040) and pVol (OR: 1.113, 95% CI: 1.013–1.223, *P* = 0.026) of Habitat 2.

### Prognostic value of tumor habitats

**Fig. 5:**
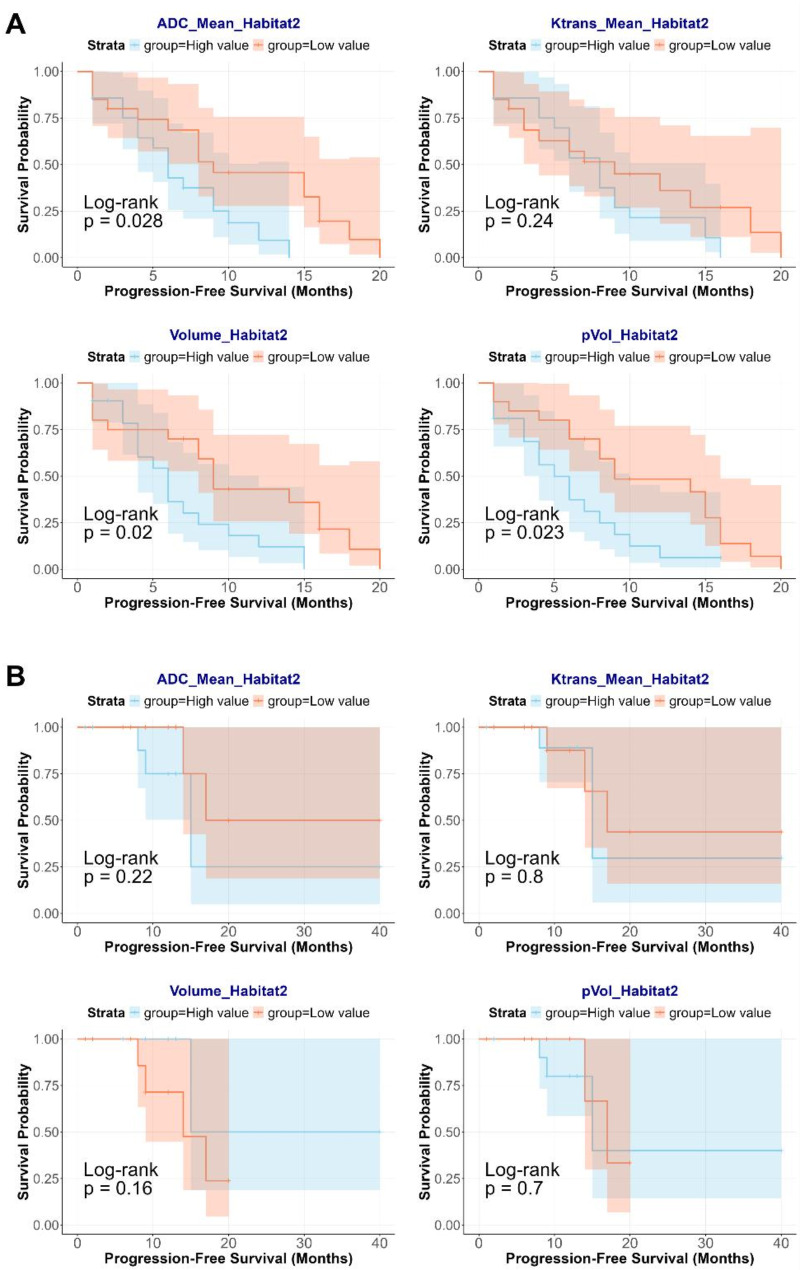
Kaplan-Meier curves for paired patient subgroups based on all quantitative metrics from Habitat 2 in (**A**) the IDH wild-type group and (**B**) the IDH mutant group.

In the IDH wild-type group, the PFS of the subgroup with high ADC_Mean in Habitat 2 was significantly shorter than that of the low-ADC_Mean subgroup in the same habitat (*P* = 0.028); patients with lower volume and pVol of Habitat 2 showed longer PFS (*P* = 0.020 and 0.023, respectively). Multivariate Cox regression revealed that only pVol_Habitat 2 was a significant risk factor for PFS (high- vs. low-pVol subgroup, HR = 2.204, 95% CI: 1.061–4.580, *P* = 0.034). The median PFS in the high-pVol_Habitat 2 (> 0.26) subgroup was 5 months shorter than that in the low-pVol_Habitat 2 (< 0.26) subgroup. Figure 5 shows the Kaplan-Meier curves for patient subgroups based on all quantitative metrics of Habitat 2. No significant PFS differences were found between paired subgroups for other habitat features in the IDH wild-type group or for any habitat features in the IDH mutant group when divided by median values (all *P* > 0.05, presented in Supplementary Fig. 3).

## Discussion

In this study, we constructed three spatial habitats for adult-type diffuse gliomas using a voxel clustering algorithm based on DWI and DCE-MRI. Features from these habitats, which characterize various intratumor cellularity and vasopermeability, showed significant differences between IDH mutant and wild-type gliomas. Multivariable logistic regression models using these features demonstrated high predictive performance for IDH genotypes. Habitat features also have prognostic value in IDH wild-type patients. These findings suggest that our habitat analysis method may provide a more precise delineation of subregions most relevant to tumor cellularity and vasopermeability in adult-type diffuse gliomas.

Intratumor molecular heterogeneity in malignant gliomas has been demonstrated in spatial transcriptome and other high-throughput sequencing research [10, 29]. On the issue of preoperatively quantifying spatial complexity and heterogeneity, spatially explicit habitat analysis methods have shown its potential [18, 30]. In our attempts, we identified three spatially distinct subregions, defined by k-means clustering of voxel-wise ADC and Ktrans parametric maps: Habitat 1 represents areas with low vascular permeability and high cellularity, where tumors may be relatively dense cell populations adapting to hypoperfusion microcirculation conditions; Habitat 2 represents areas with low vascular permeability and cellularity, likely indicating low-activity or severe-hypoxia tumor regions [7, 18, 31, 32]; Habitat 3 represents areas with high vascular permeability, likely corresponding to highly-vascularized tumors with hyper malignancy

Our results showed that the mean K^trans^ value in IDH wild-type gliomas was higher than in the mutant type within hypo-vasopermeability habitats (Habitats 1 and 2). Indeed, differences in DCE-PWI parameters between the two IDH genotypes of gliomas have been confirmed in numerous studies, and these parameters have demonstrated excellent performance in predicting IDH mutation status [33–35]. However, compared to traditional methods that focus on changes in a single parameter within the tumor region of interest, we have particularly focused on observing the spatial distribution and interactions of multiple heterogeneous biological features across the entire tumor mass. Our findings regarding K^trans^ also align with previous habitat studies, which reported increased relative cerebral blood volume (rCBV) in low-angiogenic habitats of IDH-wildtype gliomas [15, 36]. Additionally, we found that IDH mutant gliomas had a higher volume and pVol of Habitat 2, while IDH wild-type gliomas had a higher volume and pVol of Habitat 3. This suggests that there may be more cell clusters with relatively high vascular integrity in IDH mutated gliomas and more activated proliferative cell populations in IDH wild-type gliomas. Our clustering analysis showed no significant results for ADC-related habitat indices, which are consistent with previous studies based on manual VOI definition and histogram analysis [37, 38]. In fact, ADC seems to be a simplified metric influenced by many mixed histopathologic changes. Specifically, some changes may affect the signal intensity of DWI in the opposite way: high vascularity and frequent intra/extra-cellular water exchange in high-grade gliomas may increase ADC values, whereas high cellularity and macromolecular hindrance may decrease ADC values [39]. Besides, the ADC entropy, rather than ADC mean, was reported to be more resilient for reflecting IDH gene mutation status across different glioma grades [38].

Our univariate regression analysis showed that older age, the presence of necrosis and ringlike enhancement indicated a greater probability of IDH wild-type gliomas, while the frontal lobe tumors with younger age of onset and non/mildly enhanced were more likely to be the IDH mutant type. These results have been confirmed in previous clinical and morphological studies [40, 41]. The multivariable logistic regression analysis indicated that the Habitat 2 representing “hypo-vasopermeability and hypo-cellularity” was a more comprehensive habitat for characterizing differences between the two IDH genotypes, indicating a prominent variation in this subregion between IDH mutant gliomas and their wild-type counterparts. Specifically, in IDH mutant gliomas, this subregion more likely corresponds to low-malignancy tissues with relatively high vascular integrity, which may be attributed to the fact that the IDH mutation regulates downstream cytoskeletal protein (like Tau), inhibiting angiogenesis and promoting vascular normalization [7, 31]. In IDH wild-type gliomas, it is notable that this subregion appears to concentrate more around the periphery of central necrotic areas, which is consistent with histological slices and the multispectral quantification results [18, 32]. This finding suggests it more likely represents regions of severe hypoxia, which can induce epithelial-mesenchymal transition of peripheral tumors and contribute to progression and treatment resistance [42, 43]. In our entire cohort, the multivariate logistic regression model combining clinical, morphological features, and habitat metrics significantly outperformed traditional models and tumor VOI-based models in predicting IDH genotypes. Although this advantage did not overall reach statistical significance in the contrast-enhanced subgroup, our findings still highlight the potential of habitat imaging based on DCE and ADC in providing additional ITH information for predicting IDH mutation status in gliomas.

We also found that habitat features are associated with PFS in IDH wild-type patients. PFS has been pointed out as a potential surrogate endpoint for overall survival (OS) in glioblastoma, as the hazard ratios for PFS and OS were strongly correlated (*R²* = 0.92) [44]. Previous MRI-based habitat imaging studies have also observed that specific habitat indicators, such as rCBV and short-term increases of habitats reflecting perfusion and cellularity, are associated with patient survival [12, 14, 15, 36]. Interestingly, our study showed that the indicators associated with patients’ PFS in IDH wild-type gliomas were primarily found in Habitat 2, including ADC_Mean, volume, and pVol. MR spectroscopy findings on glioma intratumor metabolites have indicated that tumor regions with a high concentration of lactate, a marker of hypoxia, exhibited an increasing trend in ADC values [45]; hence the elevated ADC levels in Habitat 2 may reflect more severe hypoxia, which correlates with shorter PFS [46]. Furthermore, we found that the increased volume and pVol of Habitat 2 in IDH wild-type gliomas were associated with shorter PFS, likely due to the accumulation of severely hypoxic tissue components. All these findings seem to back up the previous hypothesis that the severely hypoxic tumor subregion is closely associated with tumor recurrence and progression. In fact, growing evidence indicates that microscopic intravascular thrombosis, induced by the neoplastic overexpression of pro-coagulants, impairs the blood supply to gliomas (resulting in perfusion-limited hypoxia), leading to extensive reorganization of the tumor microenvironment, which correlates with rapid tumor expansion, resistance to therapeutic interventions, and clinical progression [47]. Additionally, we did not observe any habitat features associated with PFS in IDH mutant gliomas. This finding may be limited by our insufficient follow-up period, and further investigation is needed for this tumor type.

Our study has some limitations. First, it was a single-center study with a small sample size. Although we validated and strengthened our findings through cross-validation and subgroup analysis, one major limitation remains the lack of internal and external validation with a larger cohort. Future studies including multi-center validations with larger sample sizes are needed. Another limitation was the inability to spatially match our images and pathological tissue samples, leading to the absence of gold standards for evaluating imaging heterogeneity. Multi-point sampling based on imaging heterogeneity maps may help address this issue and shows promise for future application. We expect that our findings may contribute to future tumor sampling strategies.

## Conclusion

In conclusion, habitat imaging derived from DCE-MRI and DWI may enable more precise delineation of subregions most relevant to tumor vasopermeability and cellularity in gliomas. Spatial habitats with distinct biological significance may provide valuable insights into intratumor heterogeneity and serve as useful imaging markers in the preoperative prediction of IDH genotypes and prognosis in adult-type diffuse gliomas.

## Electronic supplementary material

Below is the link to the electronic supplementary material.

Supplementary Material 1

## Data Availability

Data are provided within the manuscript or supplementary information files. Detailed original data from the study are available from the corresponding authors upon reasonable request.

## Abbreviations

ITH: Intratumor heterogeneity
MRI: Magnetic resonance imaging
DCE: Dynamic contrast-enhanced
DWI: Diffusion-weighted imaging
IDH: Isocitrate dehydrogenase
PFS: Progression-free survival
K^trans^: Volume transfer constant
ADC: Apparent diffusion coefficient
VOI: Volume of interest
ROC: Receiver operating characteristic
AUC: Area under the curve
