# Supplementary material for: Preoperative prediction of IDH genotypes and prognosis in adult-type diffuse gliomas: intratumor heterogeneity habitat analysis using dynamic contrast-enhanced MRI and diffusion-weighted imaging
Source: Cancer Imaging. 2025 Feb 8;25:11. doi: 10.1186/s40644-025-00829-5 (PMC11807326; doi:10.1186/s40644-025-00829-5)
Supplement: Supplementary file 1 — Supplementary Material 1 [file 40644_2025_829_MOESM1_ESM.docx]

**Preoperative Prediction of IDH Genotypes and Prognosis in Adult-Type Diffuse Gliomas: Intratumor Heterogeneity Habitat Analysis Using Dynamic Contrast-Enhanced MRI and Diffusion-Weighted Imaging**

**SUPPLEMENTARY MATERIAL**

**Figures**


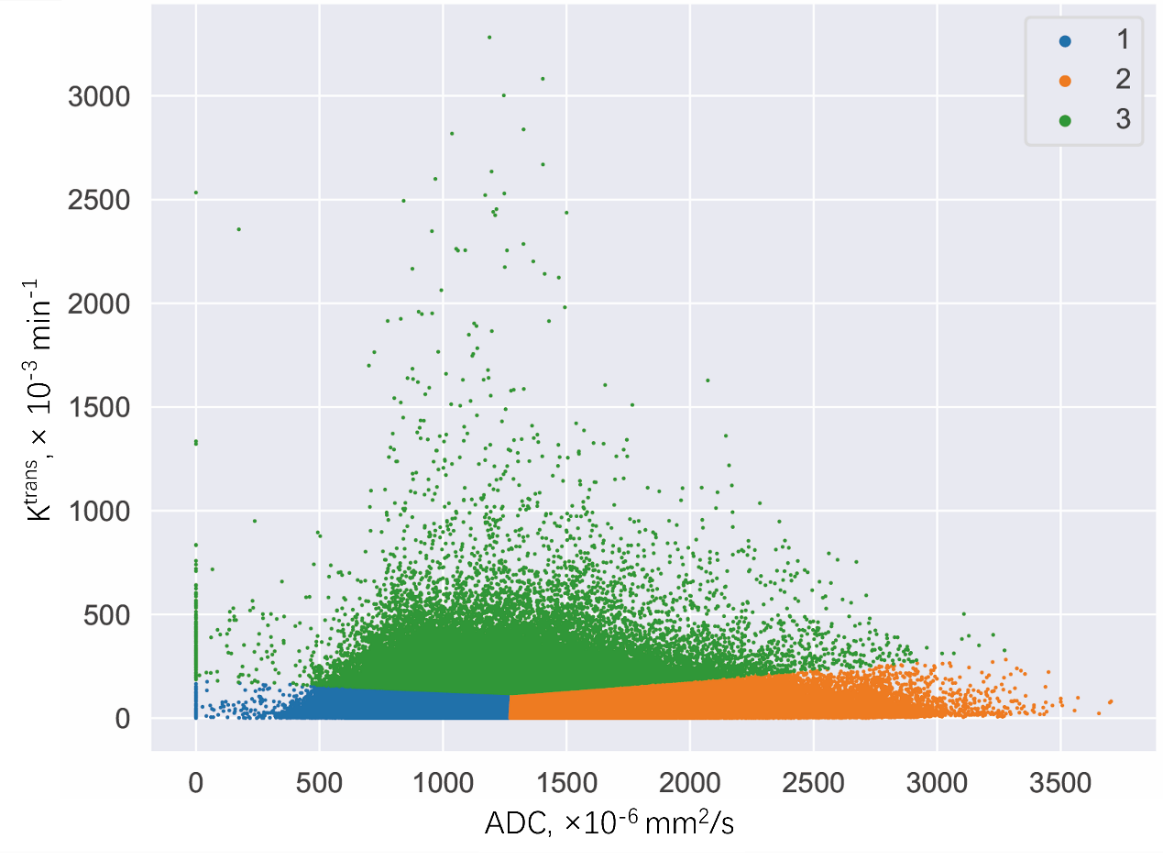


**Supplementary Fig. 1** Cohort-based demonstration of three spatial habitats defined by clustered voxels from volume transfer constant (K^trans^) and apparent diffusion coefficient (ADC) maps. Cluster 1 (blue) corresponds to Habitat 1 represents “hypo-vasopermeability and hyper-cellularity habitat” with low K^trans^ and low ADC value; cluster 2 (orange) corresponds to Habitat 2 represents “hypo-vasopermeability and hypo-cellularity” with low K^trans^ and high ADC value; cluster 3 (green) corresponds to Habitat 3 represents “hyper-vasopermeability habitat” with high K^trans^ value.


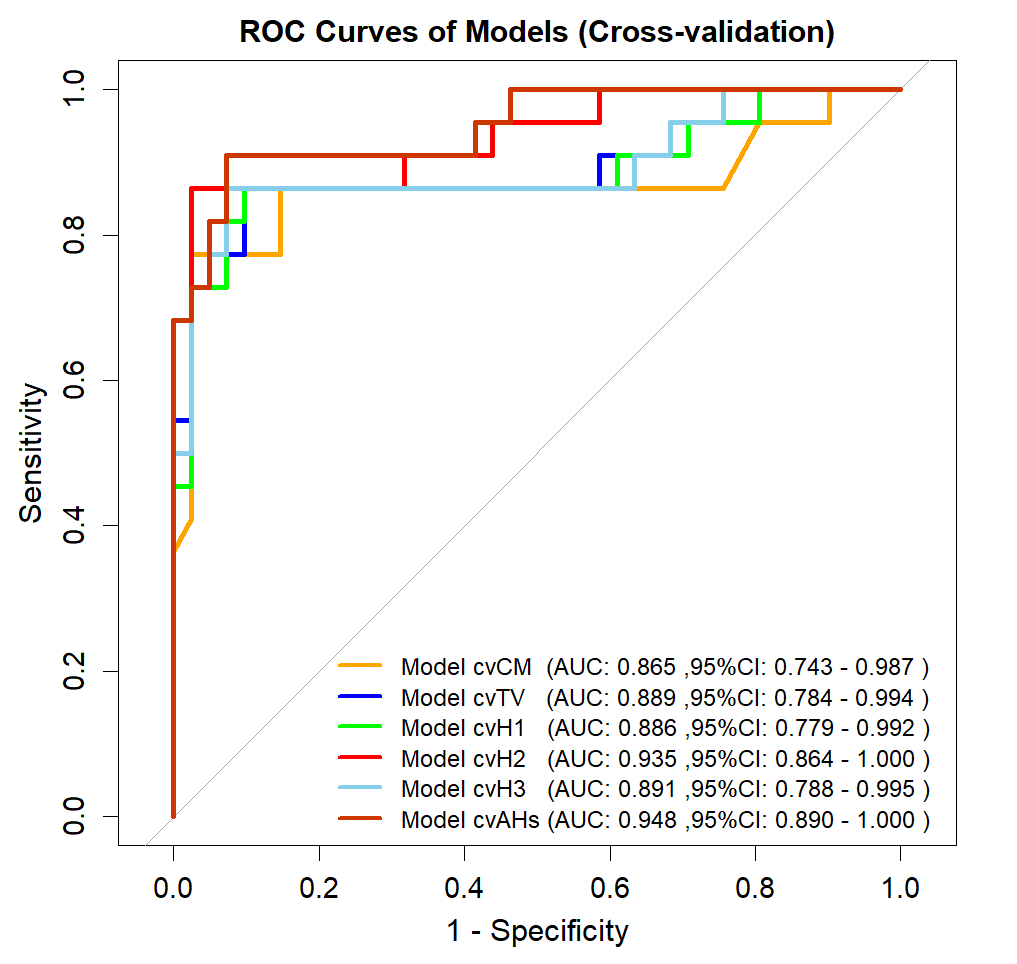


**Supplementary Fig. 2** The receiver operating characteristic (ROC) curves of the leave-one-out cross-validation for the IDH predictive models. The models cvCM, cvTV, cvH1, cvH2, cvH3, and cvAHs represent the cross-validation versions of models CM, TV, H1, H2, H3, and AHs, respectively.


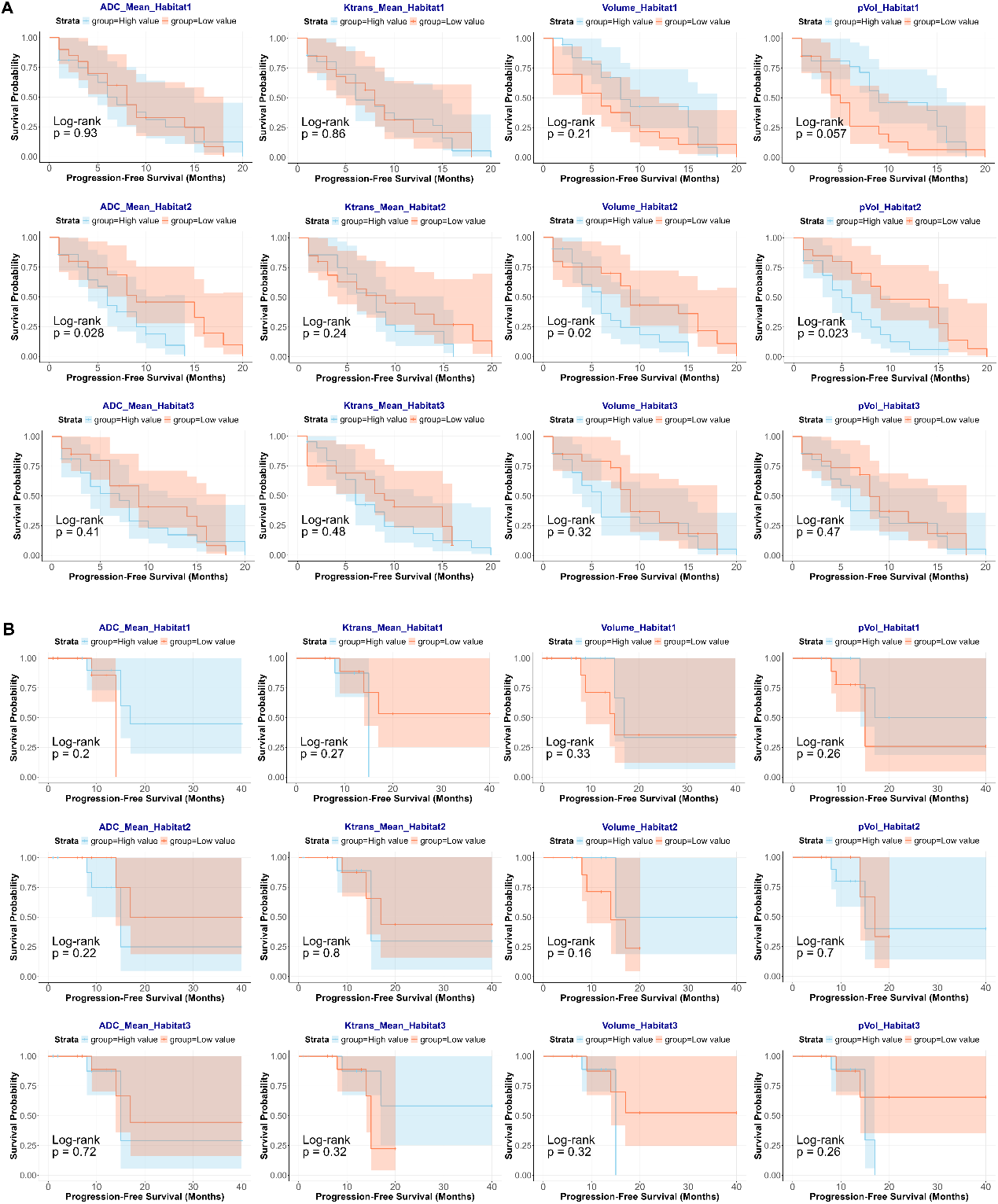


**Supplementary Fig. 3** Kaplan-Meier curves for paired patient subgroups based on all quantitative metrics from Habitats 1, 2, and 3 in (**A**) the IDH wild-type group and (**B**) the IDH mutant group.

**Tables**

| **Supplementary Table 1** Scanning parameters for MRI sequences. | | | | | |
| --- | --- | --- | --- | --- | --- |
|  | TR (ms) | TE (ms) | Matrix | FOV (mm^2^) | Slice thickness (mm) |
| DWI (b = 0, 1000 s/mm^2^) | 3300 | 87 | 384 × 348 | 199 × 220 | 5 |
| DCE-PWI | 3.86 | 1.45 | 288 × 232 | 260 × 209 | 5 |
| T1WI | 137 | 2.5 | 320 × 290 | 199 × 220 | 5 |
| T2WI | 4500 | 103 | 384 × 348 | 199 × 220 | 5 |
| FLAIR | 8000 | 86 | 320 × 290 | 199 × 220 | 5 |
| CE-T1WI | 137 | 2.5 | 320 × 290 | 199 × 220 | 5 |
| DWI, diffusion-weighted imaging; DCE-PWI, dynamic contrast-enhanced perfusion-weighted imaging; T1WI, T1-weighted imaging; T2WI, T2-weighted imaging; FLAIR, fluid-attenuated inversion recovery; CE, contrast-enhanced; TR, repetition time; TE, echo time; FOV, field of view | | | | | |

| **Supplementary Table 2** Habitat features and tumor VOI-based features in the contrast-enhanced subgroup for the two IDH genotypes (n = 51). | | | |
| --- | --- | --- | --- |
| Features | IDH+ (n = 13) | IDH- (n = 38) | *P* value |
| **Habitat 1** |  |  |  |
| ADC_Mean, ×10^-6^ mm^2^/s | 1003.79±111.53 | 1008.73±92.56 | 0.875 |
| K^trans^_Mean, ×10^-3^ min^-1^ | 37.61±20.29 | 60.02±18.19 | **0.001^*^** |
| Volume, mm^3^ | 28.79±22.42 | 26.75±18.58 | 0.747 |
| pVol (%) | 51.61±14.43 | 58.43±18.00 | 0.223 |
| **Habitat 2** |  |  |  |
| ADC_Mean, ×10^-6^ mm^2^/s | 1566.45±73.97 | 1593.20±111.10 | 0.424 |
| K^trans^_Mean, ×10^-3^ min^-1^ | 35.75±23.01 | 57.77±17.35 | **0.001^*^** |
| Volume, mm^3^ | 23.83±19.07 | 11.59±9.54 | **0.043^*^** |
| pVol (%) | 44.19±12.22 | 29.49±15.18 | **0.001^*^** |
| **Habitat 3** |  |  |  |
| ADC_Mean, ×10^-6^ mm^2^/s | 1128.38±168.71 | 1163.23±150.00 | 0.487 |
| K^trans^_Mean, ×10^-3^ min^-1^ | 190.13±21.03 | 200.85±33.48 | 0.285 |
| Volume, mm^3^ | 1.21±1.13 | 6.10±5.27 | **0.000^*^** |
| pVol (%) | 4.19±5.28 | 14.08±11.49 | **0.000^*^** |
| **Tumor VOI** |  |  |  |
| ADC_Mean, ×10^-6^ mm^2^/s | 1208.59±189.26 | 1194.73±173.30 | 0.809 |
| K^trans^_Mean, ×10^-3^ min^-1^ | 45.80±24.60 | 79.88±33.04 | **0.001^*^** |
| Volume, mm^3^ | 53.83±39.60 | 44.43±23.60 | 0.432 |
| Values are presented as mean ± SD. VOI, volume of interest; IDH, isocitrate dehydrogenase; ADC, apparent diffusion coefficient; K^trans^, volume transfer constant; pVol, volume percentage. ^*^Represented a statistical difference (*P* < 0.05) | | | |

| **Supplementary Table 3** Univariable and multivariable logistic regression analysis of IDH genotype prediction in contrast-enhanced subgroup (n = 51). | | | | | |
| --- | --- | --- | --- | --- | --- |
| Variable | Univariable | |  | Multivariable | |
|  | OR (95% CI) | *P* value | VIF^*^ | OR (95% CI) | *P* value |
| **Model CM** |  |  |  |  |  |
| Age | 0.933 (0.889, 0.979) | 0.005 | 1.117 | 0.924 (0.875, 0.975) | 0.004 |
| Frontal location | 3.927 (1.050, 14.688) | 0.042 | 1.116 |  |  |
| Necrosis | 0.229 (0.060, 0.873) | 0.031 | 1.003 | 0.160 (0.032, 0.799) | 0.025 |
| Ringlike enhancement | 0.362 (0.097, 1.358) | 0.132 |  |  |  |
| **Model TV** |  |  |  |  |  |
| Age | 0.933 (0.889, 0.979) | 0.005 | 1.126 | 0.928 (0.874, 0.985) | 0.015 |
| Frontal location | 3.927 (1.050, 14.688) | 0.042 | 1.327 |  |  |
| Necrosis | 0.229 (0.060, 0.873) | 0.031 | 1.004 | 0.138 (0.021, 0.889) | 0.037 |
| Ringlike enhancement | 0.362 (0.097, 1.358) | 0.132 |  |  |  |
| ADC_Mean | 1.000 (0.997, 1.004) | 0.804 |  |  |  |
| K^trans^_Mean | 0.963 (0.938, 0.989) | 0.005 | 1.250 | 0.959 (0.926, 0.993) | 0.018 |
| Volume | 1.011 (0.990, 1.034) | 0.307 |  |  |  |
| **Model H1** |  |  |  |  |  |
| Age | 0.933 (0.889, 0.979) | 0.005 | 1.126 | 0.924 (0.869, 0.983) | 0.012 |
| Frontal location | 3.927 (1.050, 14.688) | 0.042 | 1.242 |  |  |
| Necrosis | 0.229 (0.060, 0.873) | 0.031 | 1.008 | 0.109 (0.015, 0.808) | 0.030 |
| Ringlike enhancement | 0.362 (0.097, 1.358) | 0.132 |  |  |  |
| ADC_Mean | 0.999 (0.993, 1.006) | 0.872 |  |  |  |
| K^trans^_Mean | 0.946 (0.912, 0.981) | 0.003 | 1.161 | 0.935 (0.887, 0.986) | 0.013 |
| Volume | 1.005 (0.974, 1.038) | 0.741 |  |  |  |
| pVol | 0.976 (0.939, 1.015) | 0.222 |  |  |  |
| **Model H2** |  |  |  |  |  |
| Age | 0.933 (0.889, 0.979) | 0.005 | 1.348 | 0.924 (0.866, 0.985) | 0.015 |
| Frontal location | 3.927 (1.050, 14.688) | 0.042 | 1.399 |  |  |
| Necrosis | 0.229 (0.060, 0.873) | 0.031 | 1.023 | 0.149 (0.017, 1.332) | 0.088 |
| Ringlike enhancement | 0.362 (0.097, 1.358) | 0.132 |  |  |  |
| ADC_Mean | 0.997 (0.991, 1.004) | 0.417 |  |  |  |
| K^trans^_Mean | 0.950 (0.918, 0.983) | 0.003 | 1.550 | 0.953 (0.911, 0.998) | 0.040 |
| Volume | 1.069 (1.014, 1.128) | 0.014 | 2.018 |  |  |
| pVol | 1.092 (1.028, 1.160) | 0.004 | 1.560 | 1.113 (1.013, 1.223) | 0.026 |
| **Model H3** |  |  |  |  |  |
| Age | 0.933 (0.889, 0.979) | 0.005 | 1.188 | 0.927 (0.873, 0.984) | 0.013 |
| Frontal location | 3.927 (1.050, 14.688) | 0.042 | 1.284 |  |  |
| Necrosis | 0.229 (0.060, 0.873) | 0.031 | 1.052 | 0.193 (0.034, 1.109) | 0.065 |
| Ringlike enhancement | 0.362 (0.097, 1.358) | 0.132 |  |  |  |
| ADC_Mean | 0.999 (0.994, 1.003) | 0.479 |  |  |  |
| K^trans^_Mean | 0.987 (0.963, 1.011) | 0.284 |  |  |  |
| Volume | 0.635 (0.435, 0.927) | 0.019 | 3.401 |  |  |
| pVol | 0.865 (0.769, 0.973) | 0.016 | 3.588 | 0.859 (0.741, 0.996) | 0.044 |
| **Model AHs** |  |  |  |  |  |
| Age | 0.933 (0.889, 0.979) | 0.005 | 1.349 | **0.924 (0.866, 0.985)** | **0.015** |
| Frontal location | 3.927 (1.050, 14.688) | 0.042 | 1.426 |  |  |
| Necrosis | 0.229 (0.060, 0.873) | 0.031 | 1.024 | 0.149 (0.017, 1.332) | 0.088 |
| Ringlike enhancement | 0.362 (0.097, 1.358) | 0.132 |  |  |  |
| K^trans^_Mean_Habitat 1 | 0.946 (0.912, 0.981) | 0.003 |  |  |  |
| K^trans^_Mean_Habitat 2 | 0.950 (0.918, 0.983) | 0.003 | 2.542 | **0.953 (0.911, 0.998)** | **0.040** |
| Volume_Habitat 2 | 1.069 (1.014, 1.128) | 0.014 | 2.033 |  |  |
| pVol_Habitat 2 | 1.092 (1.028, 1.160) | 0.004 | 1.586 | **1.113 (1.013, 1.223)** | **0.026** |
| Volume_Habitat 3 | 0.635 (0.435, 0.927) | 0.019 |  |  |  |
| pVol_Habitat 3 | 0.865 (0.769, 0.973) | 0.016 | 2.180 |  |  |
| Model CM represents the multivariable logistic regression model based on clinical and morphological data. Models TV, H1, H2, and H3 are extended models based on the tumor volume of interest (VOI) and Habitats 1, 2, and 3, respectively. Model AHs is an extended model based on all habitats. IDH, isocitrate dehydrogenase; OR, odd ratio; CI, confidence interval; VIF, variance inflation factor; ADC, apparent diffusion coefficient; K^trans^, volume transfer constant; pVol, volume percentage. ^*^Variables with a value of less than 5 for this metric were finally retained before being included in the multivariable regression model. | | | | | |
